# Supplementary material for: Iron Replacement Therapy with Oral Ferric Maltol: Review of the Evidence and Expert Opinion
Source: J Clin Med. 2021 Sep 28;10(19):4448. doi: 10.3390/jcm10194448 (PMC8509126; doi:10.3390/jcm10194448)
Supplement: Supplementary file 1 [file jcm-10-04448-s001.zip › jcm-1364718-supplementary.pdf]

Schmidt et al. Iron Replacement Therapy With Oral Ferric Maltol: Review of the Evidence and Expert Opinion

Supplementary materials

Figure S1. CONSORT flow diagram.

Table S1. Designs of ferric maltol clinical trials.

Figure S1. CONSORT flow diagram.

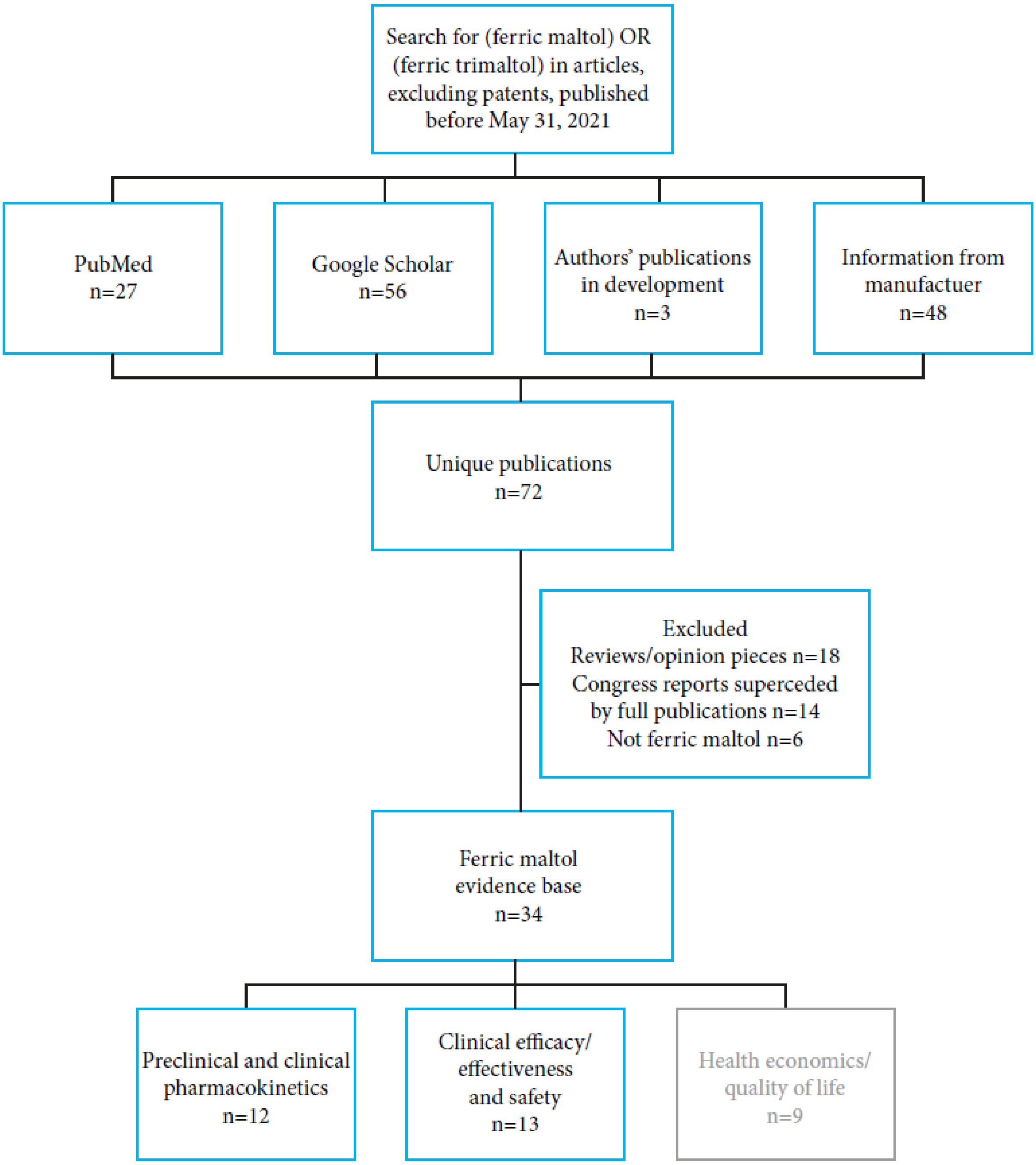

Table S1. Designs of ferric maltol clinical trials.

Study	Reference	Location	Anemia and iron deficiency definitions	Underlying condition	Population	Patients, N	Design	Comparator	Ferric maltol dosage	Treatment duration	Primary endpoint
Proof of concept	Harvey et al., 1998 [1]	UK	Hb <13 g/dL (men) or <12 g/dL (women)<br><br>Serum ferritin <15 µg/L	Gastroenterologic conditions, without active inflammation (CD n=13, UC n=2, partial gastrectomy n=2, cecal angiodysplasia n=1, and idiopathic iron-deficiency anemia following upper and lower gastrointestinal endoscopy and duodenal biopsy n=5; disease severity not specified)<br><br>Documented intolerance to 200 mg ferrous sulphate and refusal to try ferrous iron again	Not specified (presumably adult)	23	Single arm, OL, exploratory	NA	30 mg twice daily	12 weeks	Correction of anemia
Phase I IBD (adult)	Bokemeyer et al., 2017 [2]	Germany	Hb >8.5 g/dL<br><br>Ferritin <30 µg/L or ferritin <50 µg/L + TSAT <20%	IBD (CD n=13, UC n=11; disease severity not specified)	Adult	24	Randomized	NA	30, 60, or 90 mg twice daily	8 days	PK, iron uptake
Phase I (pediatric)	Allen et al., 2021 [3]	UK	Ferritin <30 µg/L or ferritin <50 µg/L + TSAT <20%	Eligibility criteria: Iron deficiency of any cause<br><br>At baseline: CD (n=8)	Pediatric	37	Randomized	NA	7.8, 16.6, or 30 mg twice daily	10 days	PK, iron uptake

				Other gastrointestinal disorders (n=11)<br>Vitamin D deficiency (n=7)<br>CKD (n=4)<br>Other conditions (n=7)							
Phase III IBD (AEGIS 1/2)	Gasche et al., 2015 [4]	Global	Hb ≥9.5 to <12.0 g/dL (women) or <13.0 g/dL (men)<br><br>Serum ferritin <30 µg/L at screening	Eligibility criteria:<br>Quiescent or mild or moderate IBD<br>UC: SCCAI score <4 at screening and randomization<br>CD: CDAI score <220 at randomization<br><br>At baseline:<br>UC: FM n=29; placebo n=29<br>Median (range) SCCAI score: FM 2.0 (0–3); placebo 2.0 (0–3)<br>CD: FM n=35; placebo n=35<br>Median (range) CDAI score: FM 75 (14–199); placebo 108 (10–220)	Adult	128 (FM n=64; placebo n=64)	Randomized, DB	Placebo	30 mg twice daily	12 weeks	Change in Hb to week 12
AEGIS 1/2 OL extension	Schmidt et al., 2016 [5]					97 entered OL phase (from DB FM n=50, from DB placebo n=47)	OL extension	NA		Up to 52 weeks, following completion of 12-week double-blind phase	Change in Hb from baseline
Phase IIIB IBD (H2H)	Howaldt et al., 2021 [6]	Global	Hb 8.0 to ≤11.0/≤12.0 g/dL (women/men)<br><br>Ferritin <30 µg/L or ferritin <100 µg/L + TSAT <20%	Eligibility criteria:<br>Quiescent or mild/moderate IBD<br>UC: SCCAI score ≤5 during screening<br>CD: CDAI score ≤300 during screening<br><br>At baseline:<br>UC:<br>FM n=46, IV FCM n=46	Adult	250<br><br>ITT: FM n=125, FCM n=125<br><br>PP: FM n=78; FCM n=88	Randomized, OL, non-inferiority	IV iron (FCM)	30 mg twice daily	12 weeks (primary endpoint), some patients treated up to 52 weeks	Hb responder rate (≥2 g/dL increase or normalization) at week 12

				Mean (SD) SCCAI score: FM 2.2 (1.8); IV FCM 2.3 (1.6)<br><br>CD:<br>FM n=79, IV FCM n=79<br>Mean (SD) CDAI score: FM 129.6 (60.1); IV FCM 140.5 (75.8)							
Phase III CKD (AEGIS-CKD)	Pergola & Kopyt, 2021 [7]	USA	Hb ≥8.0 and <11.0 g/dL<br><br>Ferritin <250 µg/L + TSAT <25% or ferritin <500 µg/L + TSAT <15%	Eligibility criteria: CKD stage III or IV (eGFR ≥15 to <60 mL/min/1.73 m^2, not on dialysis)<br><br>At baseline:<br>Mean (SD) eGFR:<br>FM 31.9 (11.5) mL/min/1.73 m^2<br>Placebo 29.7 (10.6) mL/min/1.73 m^2	Adult	167<br><br>FM n=111<br>Placebo n=56<br><br>125 started open-label FM after 16 weeks	Randomized, DB (16 weeks)<br><br>OL extension (36 weeks)	Placebo<br><br><br>NA	30 mg twice daily	16 weeks (primary endpoint) + up to 36 weeks (open label) (max total 52 weeks)	Hb change at week 16
Phase IIIB pulmonary hypertension (ORION-PH)	Olsson et al., 2020 [8]	Germany	Hb ≥7 to <12 g/dL (women) or ≥8 to <13 g/dL (men)<br><br>Serum ferritin <100 µg/L or 100–300 µg/L + TSAT <20%	Eligibility criteria: any form of PH with mean resting pulmonary artery pressure ≥25 mmHg<br><br>At baseline:<br>PAH (n=14)<br>PH due to left heart disease (n=1)<br>Inoperable chronic thromboembolic PH (n=7)<br><br>Mean (SD) pulmonary artery pressure 50 (11) mmHg	Adult	22	Single-arm, OL	NA	30 mg twice daily	12 weeks	Hb change at week 12

RWE IBD (FRESH)	Cummings et al., 2021 [9]	UK	Hb 9.5–12 g/dL (women) or 9.5–13 g/dL (men)<br><br>Serum ferritin <30 µg/L or TSAT <20%	Inactive IBD (CD n=28, UC n=28, unclassified IBD n=3)	Adult	59 (30 with primary outcome data)	RWE	NA	30 mg twice daily	12 weeks (window allowed up to 16 weeks)	Hb normalization
RWE IBD (London)	Oppong et al., 2018 [10]	UK	Not specified but presumably in line with licence	IBD (CD n=12, UC n=16; disease severity not specified)	Adult	28	RWE	NA	30 mg twice daily	Patients contacted ≥1 month after prescription	Tolerability

CD, Crohn's disease; CDAI, Crohn's Disease Activity Index; CKD, chronic kidney disease; DB, double-blind; eGFR, estimated glomerular filtration rate; FCM, ferric carboxymaltose; FM, ferric maltol; H2H, head-to-head; Hb, hemoglobin; IBD, inflammatory bowel disease; ITT, intention-to-treat; IV, intravenous; NA, not applicable; OL, open-label; PAH, pulmonary arterial hypertension; PH, pulmonary hypertension; PK, pharmacokinetics; PP, per protocol; RWE, real-world evidence; SCCAI, Simple Clinical Colitis Activity Index; SD, standard deviation; TSAT, transferrin saturation; UC, ulcerative colitis.

References

1. Harvey, R.; Reffitt, D.; Doig, L.; Meenan, J.; Ellis, R.; Thompson, R.; Powell, J. Ferric trimaltol corrects iron deficiency anaemia in patients intolerant of iron. *Aliment Pharmacol Ther* **1998**, *12*, 845-848, doi:10.1046/j.1365-2036.1998.00380.x.
2. Bokemeyer, B.; Krummenerl, A.; Maaser, C.; Howaldt, S.; Moß, M.; Mallard, N. Randomized open-label phase 1 study of the pharmacokinetics of ferric maltol in inflammatory bowel disease patients with iron deficiency. *Eur J Drug Metab Pharmacokinet* **2017**, *42*, 229-238, doi:10.1007/s13318-016-0334-5.
3. Allen, S.; Auth, M.K.-H.; Kim, J.J.; Vadamalayan, B. Safety, tolerability and pharmacokinetics of oral ferric maltol in children with iron deficiency: Phase 1 study. *JPGN Rep* **2021**, *2*, e090, doi:10.1097/PG9.0000000000000090.
4. Gasche, C.; Ahmad, T.; Tulassay, Z.; Baumgart, D.C.; Bokemeyer, B.; Büning, C.; Howaldt, S.; Stallmach, A. Ferric maltol is effective in correcting iron deficiency anemia in patients with inflammatory bowel disease: Results from a phase-3 clinical trial program. *Inflamm Bowel Dis* **2015**, *21*, 579-588, doi:10.1097/MIB.0000000000000314.
5. Schmidt, C.; Ahmad, T.; Tulassay, Z.; Baumgart, D.; Bokemeyer, B.; Howaldt, S.; Stallmach, A.; Büning, C.; Group, A.S.; Baumgart, D.C. Ferric maltol therapy for iron deficiency anaemia in patients with inflammatory bowel disease: Long-term extension data from a Phase 3 study. *Aliment Pharmacol Ther* **2016**, *44*, 259-270, doi:10.1111/apt.13665.

6. Howaldt, S.; Domènech, E.; Martinez, N.; Schmidt, C.; Bokemeyer, B. Long-term effectiveness of oral ferric maltol versus intravenous ferric carboxymaltose for the treatment of iron-deficiency anemia in patients with inflammatory bowel disease: A randomized controlled noninferiority trial. *Inflamm Bowel Dis* **2021**, May 14, 2021 (epub ahead of print), doi:10.1093/ibd/izab073.
7. Pergola, P.E.; Kopyt, N.P. Oral ferric maltol for the treatment of iron-deficiency anemia in patients with chronic kidney disease: Phase 3, multicenter, randomized, placebo-controlled trial and open-label extension. *Am J Kidney Dis* **2021**, May 21, 2021 (epub ahead of print), doi:10.1053/j.ajkd.2021.03.020.
8. Olsson, K.M.; Fuge, J.; Brod, T.; Kamp, J.C.; Schmitto, J.; Kempf, T.; Bauersachs, J.; Hoeper, M.M. Oral iron supplementation with ferric maltol in patients with pulmonary hypertension. *Eur Respir J* **2020**, *56*, 2000616, doi:10.1183/13993003.00616-2020.
9. Cummings, J.F.; Fraser, A.; Stansfield, C.; Beales, I.; Sebastian, S.; Hoque, S. Ferric maltol Real-world Effectiveness Study in Hospital practice (FRESH): Clinical characteristics and outcomes of patients with inflammatory bowel disease receiving ferric maltol for iron-deficiency anaemia in the UK. *BMJ Open Gastroenterol* **2021**, *8*, e000530, doi:10.1136/bmjgast-2020-000530.
10. Oppong, P.; Lovato, S.; Akbar, A. PTU-124 Real world tolerability & efficacy of oral ferric maltol (feraccru) in IBD associated anaemia. *Gut* **2018**, *67*, A252-A253, doi:10.1136/gutjnl-2018-BSGAbstracts.502.
